# The Influence of Age, Sex and Season on Andean Condor Ranging Behavior during the Immature Stage

**DOI:** 10.3390/ani13071234

**Published:** 2023-04-02

**Authors:** Jorgelina María Guido, Nicolás Rodolfo Cecchetto, Pablo Ignacio Plaza, José Antonio Donázar, Sergio Agustín Lambertucci

**Affiliations:** 1 Grupo de Investigaciones en Biología de la Conservación (GRINBIC), Laboratorio Ecotono, INIBIOMA (Universidad Nacional del Comahue–CONICET), Pasaje Gutiérrez 1125, San Carlos de Bariloche R8400FRF, Río Negro, Argentina; 2 The Peregrine Fund, 5668 West Flying Hawk Lane, Boise, ID 83709, USA; 3 Grupo de Ecología de la Polinización (EcoPol), Laboratorio Ecotono, INIBIOMA (Universidad Nacional del Comahue–CONICET), Pasaje Gutiérrez 1125, San Carlos de Bariloche R8400FRF, Río Negro, Argentina; 4 Department of Conservation Biology, Estación Biológica de Doñana, CSIC, 41092 Sevilla, Spain

**Keywords:** dispersal, flight distance, home range, movement patterns, scavengers, *Vultur gryphus*, vultures

## Abstract

**Simple Summary:**

To better understand animals’ ecology, mitigate threats and protect species effectively, it is necessary to know how all age and sex categories use the space over time. However, little is known about how many immature animals move throughout their dispersal period. Here, we describe the movement patterns of immature Andean condors during the immature stage, analyzing whether these movements differ according to age, sex and season. We found that immature condors display the longest home ranges and flight distances during warm seasons and when they are sub-adults. Males tend to have larger home ranges than females. The movement patterns we found were larger than those reported for adult condors, but also much larger than those reported for immature individuals from other vulture species. We highlight the importance of understanding and considering immature individuals’ movements, the area they use and their capabilities of movement in conservation strategies.

**Abstract:**

Immature individuals move from their natal area to the area where they settle and reproduce, and this may take several years. This process is essential for long-lived species such as vultures and condors, which spend long periods as immature and move extensively. We studied the movement behavior of 26 GPS-tagged immature Andean condors (*Vultur gryphus*) from northwestern Patagonia throughout the immature stage, analyzing whether these patterns differed according to age, sex and season. We found that season and age influenced home range size and flight distances, the warm season being when immature condors move most; movement patterns were greater in sub-adults than in juveniles. The age effect was associated with the sex of individuals, with males increasing their home range more than females. Our results provide the first description of how immature Andean condor movement patterns are affected by internal and external factors. This information could be key to understanding condor responses to environmental change and threats at different stages during their immature phase. Until now, condor conservation efforts have not considered the areas used by dispersing individuals. Our results increase our understanding of ranging behavior during the immature stage of this threatened bird, enabling us to improve the conservation policies and management strategies designed to protect them.

## 1. Introduction

The immature phase is an important stage; it is when individuals disperse, and learn how to find food and other resources for surviving. During this phase, individuals disperse, leaving their natal area until they settle and reproduce for the first time [1,2,3]. This phase is not only important at an individual level, but also for population dynamics, because immature individuals evaluate the spatiotemporal distribution of resources, which is essential for species that live in changing environments [2,3]. In the past, most movement studies were based on direct field observations of marked animals, or on very high frequency (*VHF*) radio-telemetry tracking of individual animals (e.g., [4,5,6,7,8]). This has often resulted in inaccurate and biased information, especially for wide-ranging species that can move hundreds of kilometers in a single day. In fact, due to the difficulty of studying movement patterns during the immature stage, a large knowledge gap still exists in our understanding of this period [9,10,11].

Over recent decades, satellite tracking technology has produced high-quality information about animal movement and home range areas [9,12]. This technology has improved the information available on habitats that are important for wild animals, especially for long-lived species [11,13]. Moreover, satellite tracking has clearly showed that even within a species there may be important differences in the use of space, depending on age, sex and seasonality [14,15,16,17]. Satellite tracking has also made it possible to improve our knowledge on dispersal in several animal species [1,18,19], particularly in immature birds [6,20,21,22]. However, information is scarce on the immature stage of birds of conservation concern, such as vultures and condors (e.g., [9]).

Studying the immature phase of large soaring birds can provide new insights into bird movement, since their life stories differ from those of other, frequently-studied birds such as owls and passerines [21,23,24,25,26]. Most soaring birds are long-lived and have a low reproductive rate, with prolonged parental care [27]. Their offspring present delayed maturity, dispersal being a long and complex process during which immature individuals have to learn how to be self-sufficient, acquire flight skills, find food resources and exploit them efficiently [28,29]. Using their capacity for extensive movement, vultures have greater dispersal potential than other birds [15,30,31], and studying this process may reveal important new information for their conservation. For example, key sites for vulture conservation can be identified (e.g., resting sites such as communal roosts or risky flight paths commonly used by immature birds but less used by adults) [11,13,32]. For these reasons, studying immature vultures and condors is of great importance in creating effective conservation policies for these threatened birds.

The Andean condor (*Vultur gryphus*), an emblematic species of South America, is facing diverse threats associated with human disturbance (e.g., lead contamination, intentional poisoning, persecution and habitat loss) [33]. Despite being considered a Vulnerable species [34], almost no information exists on the movement patterns and differential range use of immature individuals (however, see [35]). In this study, therefore, we provide an integrated description of the movement behavior of immature Andean condors at different moments of their dispersal period (from the first months after leaving the nest, ≥1year old, up to several months before settling in a territory, 6 years old), analyzing whether these patterns differ according to age (juvenile or sub-adult), sex and season (warm or cold). This analysis of movement during the immature stage improves our understanding of condor population dynamics and can be used to improve the conservation measures that focus on this species.

## 2. Materials and Methods

### 2.1. Study Area

The study was performed in northwestern Patagonia, Argentina and Chile (33–50.5° S/68–72.5° W). This area was determined by the movement patterns of the tagged Andean condors. This area includes a gradient of high mountain environments in the west, and temperate forests, pastures and sub-Andean steppes in the east [36]. The Andes Mountains become flatter to the east, turning first into a transitional area (‘ecotone’) and then into the Patagonian Monte (in the north) and the Steppe (in the south) biomes [36,37]. Condors commonly use the ecotone and the Steppe to feed on livestock and wild herbivores [38,39,40]; the mountains between Chile and Argentina are used for nesting and roosting [39].

### 2.2. Study Species

The Andean condor is a large obligate scavenger that inhabits the Andes Mountain range in South America (from Venezuela to the south of Chile and Argentina) [34,41]. Condor populations have suffered a retraction in some areas, and their populations are tending to decrease [33,34,42]. Their reproductive strategy is one of the slowest among birds; they commonly lay one egg every two years, then the chick spends up to six months in the nest, and stays with its parents until 15 months of age [27,43]. They also have a long immature period that takes up to six years [41,44]. This species is among the largest flying birds in the world, with a wingspan that can exceed 3 m, and notable sexual dimorphism that allows differentiation between sexes: males have a crest and are bigger than females, weighing up to 16 kg [27,45].

### 2.3. Bird Tagging

We trapped Andean condors in the surroundings of Bariloche city, Río Negro province, Argentina (41°13′ S, 71°4′ W). For this, we used a cannon net baited with a sheep carcass. During the austral spring–summer seasons between 2013 and 2018, we trapped 26 immature Andean condors (14 females and 12 males) with an age range of between two and five years old. Individuals were assigned to age categories according to their bill and feather coloration: juvenile (post-fledgling to 3 years) or sub-adult (3 to 6 years) [44,46,47]. We tagged 17 of the trapped birds with a 100-g solar GPS-GSM CTT backpack (Northstar-VektorTek LLC, Reston, VA, USA), four with a 90-g solar CTT^®^-1090 GPS-GSM backpack (Cellular Tracking Technologies, Rio Grande, NJ, USA), and five with a 75-g solar CTT^®^-1000-BT3-Series GPS-GSM 3rd Gen backpack (Cellular Tracking Technologies) (Table S1). The devices weighed between 75 and 100 g (<1.5% of the weight of the tagged birds) and were fitted with Teflon ribbon backpack harnesses. All the tags were duty cycled to transmit every day from dawn to dusk at the minimum interval time allowed by the tags, i.e., every 15 min.

### 2.4. Data Analysis

#### 2.4.1. Data Processing

We obtained 249,101 GPS locations from the 26 tagged immature condors, registered from the time each bird was released until the time the unit stopped working or up to 1 December 2020. Only the devices placed in 2018 are still transmitting, the others having failed at different times, so the monitoring periods differed between individuals (Table S1). Unfortunately, we do not know with confidence the causes for the failure of the units; however, those failures are frequent in telemetry studies [48]. To standardize the interval time between successive GPS locations, and to use a time interval comparable with the available information for adult Andean condors and immature individuals of other vulture species, we used a subsample of our dataset that included only one GPS location per hour. For all spatial analyses, the GPS locations were projected to the UTM coordinate system (WGS-1984 UTM Zone 19S) for use in R v.3.6.0 [49] and ArcGIS v.10.3 (ESRI, Inc. USA). For all temporal analyses, we compared two seasons based on the number of daylight hours: “warm” (1 October–31 March) and “cold” (1 April–31 September). For all spatial analyses involving seasonal comparisons, we used only data from individuals that registered at least 140 GPS locations in an entire month; this value represents the asymptotic value of the monthly home range curves for most of the immature individuals surveyed.

#### 2.4.2. Home Range Size

To describe the area used by all the tagged immature Andean condors and to estimate the total and monthly home range, we assessed a combination of home range estimators using all the GPS locations obtained for each individual [50,51]. For the statistical analyses, however, we used the locations of each individual in each month, taking into account the season of each dataset, and the age and sex of each bird. Home ranges were estimated through kernel density estimation (KDE) using the “adehabitatHR” and “rgal” packages in R software [49,52,53]. Kernel density estimator (KDE) contours were also calculated to report the total area used (99%), the majority of the home range areas (95%), and the core (intensive use) areas (50%). We applied a smoothing parameter (h) of 7000 and a grid size of 10,000 m following the ad hoc criteria suggested by [54]. We also calculated the home range of each individual with the 100% Minimum Convex Polygon (MCP) encompassing all GPS fixes obtained for that individual. Although MCPs tend to overestimate the actual area occupied by an individual, they provide an indication of the overall foraging area and allow comparisons with other studies [9,55,56].

#### 2.4.3. Flight Distances

To quantify the extent of immature Andean condor movements, we determined the distance traveled in hourly fixed intervals for individuals across all classes of age, sex and season. Hourly flight distances were calculated as the distance in a straight line between two consecutive locations registered with one hour’s difference on the same day. Daily distances were calculated as the sum of the distances from all locations in consecutive hours in a single day for each individual. For the statistical analyses and to avoid the underestimation of daily flight distances due to the loss of GPS locations, we used only the days that had data for at least 75% of the possible hourly locations, considering the maximum available hours of daylight per day. For warm seasons the maximum number of daily hours was 16, whereas in cold seasons it was 12 h [45]. Therefore, in the warm season, only days with at least 12 locations (i.e., between 12 and 16 locations) on the same day were considered, whereas in the cold season only days that had data for at least 9 locations (i.e., between 9 and 12 locations) on the same day were considered.

### 2.5. Statistical Analysis

To estimate home range size, we worked with a set of monthly data grouped into warm or cold seasons. We also considered the potential effect of age, sex, season and the interactions between these variables on home range size and distance traveled. As mentioned above, we included in the analysis only individuals that had at least 140 GPS locations per month; that is, individuals that reached the monthly home range asymptote (Table S2 and Table S3). To evaluate how individual movements can be affected by age (juvenile or sub-adult), sex and season (warm or cold), we use mixed effect models fitted to data using the maximum likelihood estimation [57]. We applied log transformations to the movement variables to reach the model assumptions. The logs of (i) home range size (MCP, KDE 99%, KDE 95%, KDE 50%) and (ii) hourly and daily flight distances were included as response variables. We considered the interactions between age, sex and season variables as predictors. As triple interactions were never significant, we included only double interactions as fixed effects in the models. Since home range size and flight distance can be influenced by an individual’s intrinsic characteristics and the year, the identity of each individual (ID) and the year were included in the models as random effects. The analyses were performed using the “nlme” R software package [49,58].

## 3. Results

### 3.1. Home Range Size

**Figure 1:**
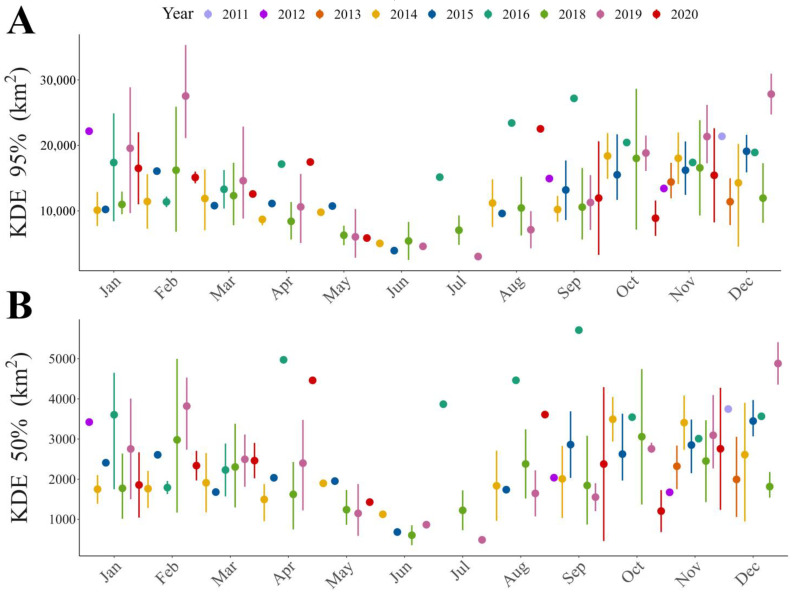
Trend throughout the year of home range size for different home range estimators: (**A**) Kernel Density Estimator (KDE) of 95% and (**B**) Kernel Density Estimator (KDE) of 50% of the contour area.

Taken together, all the immature condors used an area of 438,260 km^2^ MCP (KDE 99% = 206,854.7 km^2^; KDE 95% = 121,812.1 km^2^; KDE 50% = 15, 282.9 km^2^). The marked birds covered a large part of the southern mountain range; in a north–south direction their range extended 1969.7 km, and in a west–east direction the maximum covered was 385.4 km. We obtained a total of 65,190 GPS locations per hour from the 26 immature GPS-tagged Andean condors. However, for the analysis of home range size, we used 54,091 GPS locations of 18 individuals: 8 females and 10 males that reached the asymptotic value of the home range curves. Five juvenile individuals were also monitored for a short time period during their sub-adult stage (Table S2). Home range size varied between individuals (Figure 1; Table S2; Figure S1). The largest home range was 227,162.4 km^2^ MCP (Table S2), and was recorded in a sub-adult male condor (ID: TOTO). The maximum home range estimated by kernel was 114,995.2 km^2^ KDE 99%; see other kernel estimations in Table S2.

**Figure 2:**
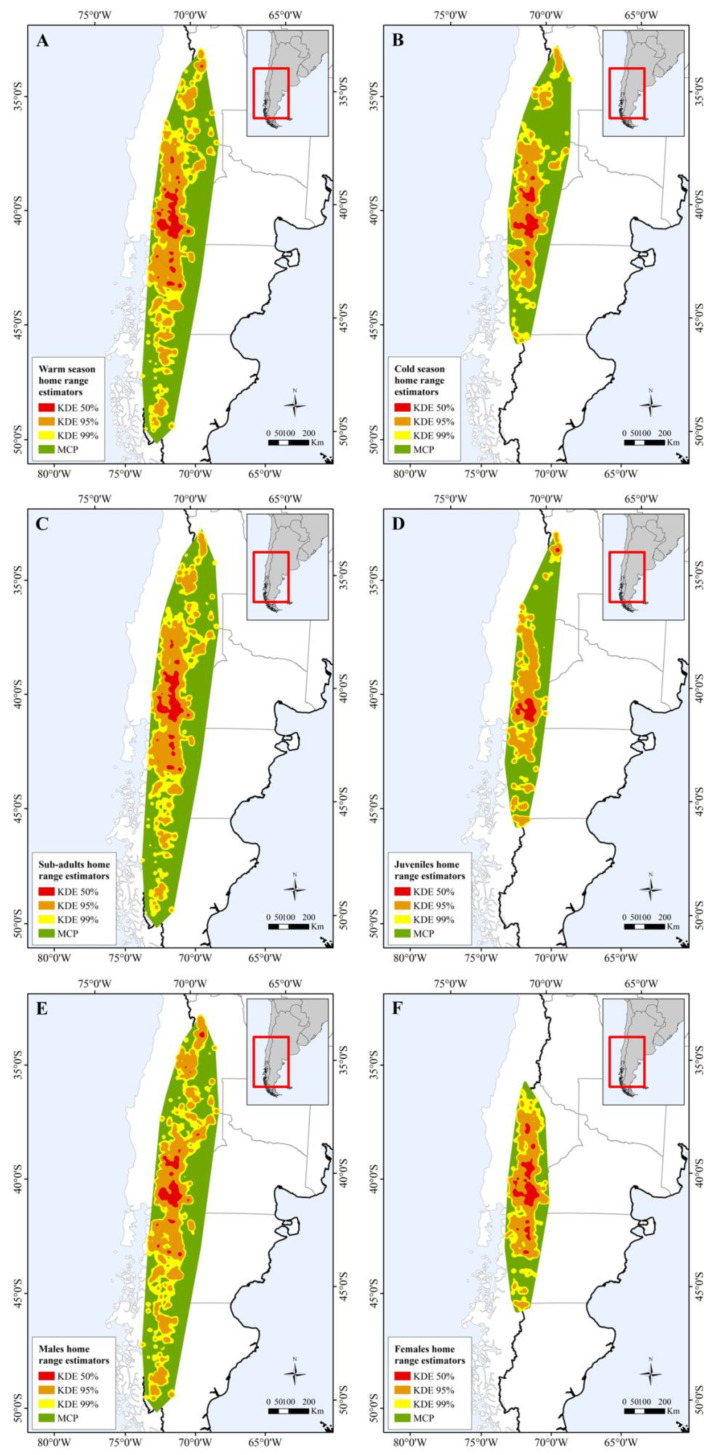
Map showing differences in home range size between: (**A**) warm and (**B**) cold seasons; (**C**) sub-adults and (**D**) juveniles; and (**E**) male and (**F**) female individuals.

**Figure 3:**
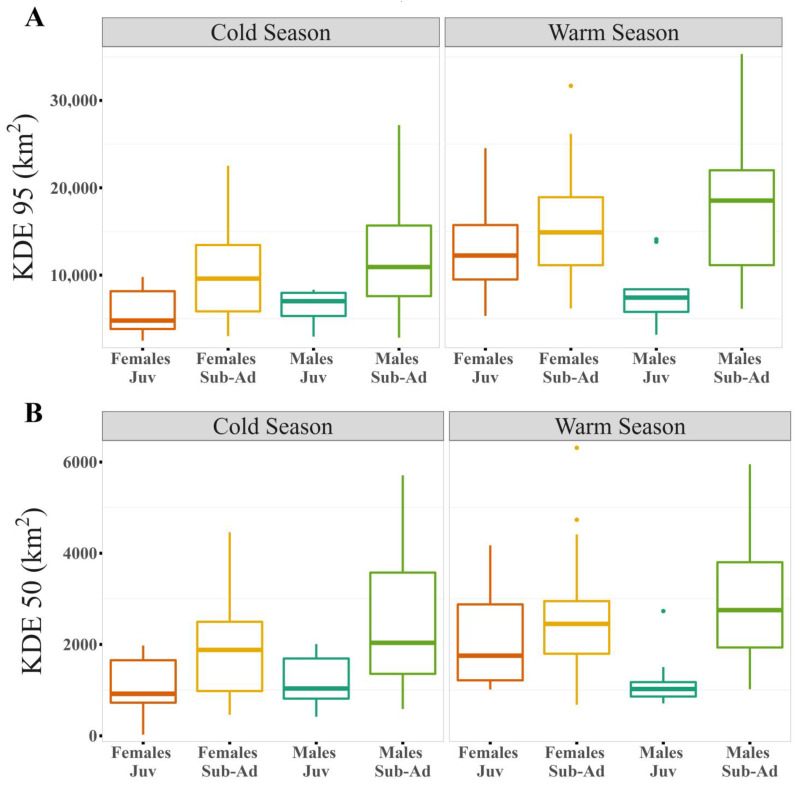
Differences between sexes and ages by season for the logarithm of the different estimators: (**A**) Kernel Density Estimator (KDE) of 95% and (**B**) Kernel Density Estimator (KDE) of 50% of the contour area.

Our results show that home range size differs between seasons (Table 1 and Table S4), regardless of the estimator used. The home range was larger during warmer months than during colder months (Figure 1, Figure 2A,B and Figure S1). During the warm season, the maximum home range found was for a sub-adult male (ID: HECTOR; KDE 95% = 80,087.58 km^2^; KDE 50% = 10,027.22 km^2^), while in the cold season the maximum value was for a juvenile female (ID: BM27B3_b; KDE 95% = 40,265.4 km^2^; KDE 50% = 6148.2 km^2^). Our results also suggest that age affects the MCP and KDE 99% home range (Table S4), with sub-adults presenting larger home ranges than juveniles (Figure 2C,D, Figure 3 and Figure S2). However, when using kernel density estimators for the majority and most used areas, i.e., 95 and 50% of the contour area, this effect depended on the sex of the individuals (Figure 2E,F, Figure 3 and Figure S2; Table 1), with sub-adult males having the largest home range size (Table 1).

### 3.2. Flight Distances

**Figure 4:**
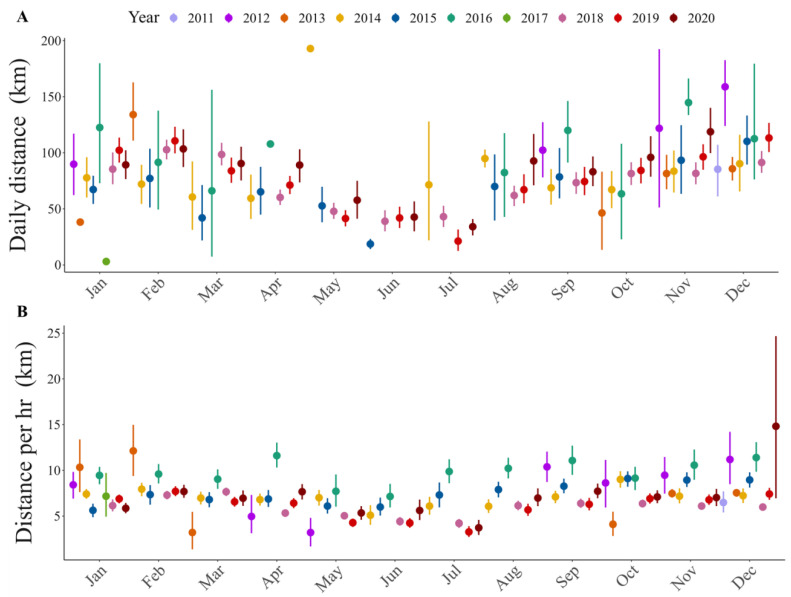
Trend throughout the year of flight distance: (**A**) per day, (**B**) per hour.

To estimate hourly flight distances, we obtained 55,871 GPS locations registered at one-hour intervals. Flight distances were variable between individuals (Figure 4; Table S2). The maximum distance flown in one hour was 118.7 km, which was recorded in a sub-adult male condor (ID: HECTOR) during the warm season. Results show that hourly flight distances differ between ages (Table 2), with sub-adults showing greater hourly distances than juveniles. Our results also show that the seasons affect the hourly flight distance, but this effect depends on the sex of the individuals (Table 2). During warm seasons, males fly the greatest distances in one hour.

From the hourly flight distances obtained, we estimated 2581 daily distances. The greatest daily distance flown by an immature condor was 302.2 km and was recorded in a sub-adult male condor during the warm season (ID: HECTOR) (Table S2). The mean daily distance flown was 80.5 km for all the condors, and 93.7 km was the highest mean distance in an individual, recorded in a sub-adult male condor (ID: CT4072). Daily flight distances also differed between seasons (Table 2): the daily distances flown were greater during warm seasons than cold ones. Moreover, we found differences between ages in the daily distances flown (Table 2); sub-adult Andean condors made the longest flights.

## 4. Discussion

We found that immature Andean condors can cover huge areas in flight (up to 438,000 km^2^, based on MCP home range size estimation) during their immature phase. They also have an impressive ability to cover large areas in short time periods, being able to travel almost 120 km in an hour and more than 300 km in a day. Only one bird covered a distance of 1350 km north–south in a dispersal period of 33 months; all the tagged birds (marked in the same place) covered almost 2000 km north–south, which is equivalent to the distance from Madrid (Spain) to Rome (Italy) or more than the distance from Lima (Peru) to Quito (Ecuador), as examples.

Most animal species present spatial segregation biased by age, mainly as a consequence of reproductive status and differences in how they exploit resources [13,14,17,32]. Previous studies on adult Andean condors reported a maximum home range size of 77,309.2 km^2^, calculated by MCPs, and 41,783.1 km^2^ for the KDE of the 95% contour area [59]. In our study, the maximum MCP value for an immature condor was almost three times higher than this (227,162.4 km^2^), and almost twice as large in the case of the KDE 95% (72,668.1 km^2^). Our results also show that sub-adult condors have larger home range sizes and perform longer hourly and daily flights than juveniles. However, despite having a smaller home range, adult condors may fly greater distances each day (maximum distance: 349.5 km; mean distance: 152.3 km for the individual who flew the most) [60] than immature condors (maximum distance: 302.2 km; mean distance: 93.7 km for the individual who flew the most).

### Condors vs. Other Vulture Movements

Differences in movement patterns between the adults and immature individuals of a species may be explained by the fact that, during the dispersal period, immature birds usually move more freely, with long trips and erratic movements [1,13]. They explore new areas, searching for resources without the need to return to the same place (i.e., they are not tied to a specific territory, such as a breeding area). These differences can also vary within a category, so the age of the immature birds can also affect their movement patterns [13,17]. Immature Andean condor movement patterns are influenced by age: sub-adult condors have larger home range areas and make longer flights than juveniles. These results agree with movement patterns reported in other vulture species. For example, during the first months of life, fledgling Bearded (*Gypaetus barbatus*) and Cape vultures (*Gyps coprotheres*) present small home ranges and make short flights; with time, they increase the territory they explore [30,61,62]. Therefore, sub-adult individuals fly large distances, increasing the size of the area used until they became adults [14,15,63,64,65].

The sex of individuals is another factor influencing animal dispersal during the immature stage [2,66,67]. In birds, it is common that females disperse more frequently and farther than males [2,67]. However, this difference has not been reported for vultures, probably because they are generally monomorphic species [14,68]. However, in a strongly dimorphic, socially complex species such as the Andean condor, an individual’s use of space can be affected by its position in the despotic social system. Condors have marked hierarchies established by sex and age, resulting in an asymmetric pattern of habitat use and food exploitation [47,69,70]. In this sense, sub-adult males may be perceived by adults as possible competitors, so they could be forced to disperse early from their natal territories or from the best foraging and resting areas, in contrast to females and juveniles [71]. Moreover, more tolerance is shown towards females, particularly under adverse conditions, so females can remain close to natal territories [67,71].

Our results suggest that seasonality has a marked effect on immature Andean condor movements; during the warmer season, these birds moved more than during the colder one. The most notable reductions occurred between May and July (winter season in the Southern Hemisphere). Temporal variations in animal movements can be due to internal and external factors [72,73]. Common examples of internal and external factors are the reproductive period and the weather conditions, respectively. Bearded vultures begin the dispersal period at the same time as a new adult’s reproductive period; at this time, fledglings begin to make longer flights [30,61]. Similarly, sub-adults increase their movements during this period, as adults are more prone to defend their territories, forcing them to undertake longer foraging and exploratory movements [14]. The weather is widely considered in vulture movement studies, variations in movements being analyzed according to the season (e.g., [14,74,75,76,77]). In general, summer days are longer and warmer than winter days, improving flight conditions for soaring bird species like these [68,76,78].

The immature individuals we monitored had huge home ranges that even exceeded the current records for other vultures worldwide (e.g., [15,32,65,79]). Among non-migratory species, the maximum value of MCP found was for a sub-adult Bearded vulture (51,620 km^2^) [14]; this maximum value only represents 22.7% of the maximum value found in an immature Andean condor (227,162 km^2^). Moreover, among the non-migratory vulture species, the maximum daily flight distance found was 267.4 km for a sub-adult White-backed vulture [65]; this value is 35 km less than the maximum value estimated for a sub-adult Andean condor (302.2 km) in this study. To the best of our knowledge, among immature vultures Andean condors showed the highest values for both home range and daily flight distances. This could be because Andean condors in Patagonia inhabit regions where they depend on extensive livestock ranching and wild species, so they need to cover large areas when foraging [39,40]. Moreover, there has been a decrease in marine fauna on the Chilean coast, which in the past served as food for these birds. This may oblige them to make longer trips in search of food—many birds from Chile cross the Andes Mountains between the two countries to feed on the Argentine Patagonian steppe, increasing competition for food [39].

## 5. Conclusions

In this study we describe, for the first time, the movement patterns of immature Andean condors, which are the most extensive so far reported for vultures worldwide. This highly valuable information is necessary for a better understanding of the movement ecology of the species, and its association with, for instance, anthropogenic threats [33]. It is also relevant to the design of management strategies and conservation action [35,42,80]. The information provided can also be useful for other species that cross national and international borders, given that conservation action and management policies often differ between countries [60,81]. Our results suggest that immature condors can fly hundreds of kilometers in a single day, even crossing national and transnational boundaries during each flight. This exposes them to multiple threats, as the proportion of time spent in protected areas is reduced [35,80]. It is evident that protecting this species presents a great conservation challenge due to its wide-ranging behavior.

Although much is being done to conserve Andean condors throughout their entire distribution, their populations continue to decline [33]. Several factors are driving this population decline, but the most important seem to be those associated with human activities that directly or indirectly affect the birds’ survival (e.g., poisoning) [33,82]. Previous studies have shown the diverse threats this species suffers; however, these articles often focus on adult individuals (e.g., [45,60,81]). This limits recommendations that can be made for areas of special interest for the conservation of this species, since these recommendations only consider the most stable portion of the population, whose movements are more tied to a territory than those of immature birds. Considering immature individual behavior within management plans is crucial for effective protection of the reproductive potential of the species. Taking the movement patterns of immature populations into account in conservation strategies may improve species management; this is particularly relevant for populations that are threatened and in decline.

## Figures and Tables

**Table 1:** Linear mixed models that evaluate how the home range size Kernel Density Estimator (KDE) of 95% and 50% of the contour areas of immature Andean condor individuals can be affected by age (juvenile or sub-adult), sex, season (warm or cold) and the interactions between these variables. The asterisks show statistically significant results.

Model	Variables	Est. Value	Lower	Upper	Std. Error	*t*-Value	*p*-Value	
KDE 95%	(Intercept)	3.843	3.695	3.991	0.076	50.460	0.000	*
	Season (warm)	0.243	0.110	0.377	0.069	3.530	0.001	*
	Age (sub-adult)	0.069	−0.108	0.245	0.086	0.796	0.435	
	Sex (male)	−0.094	−0.304	0.116	0.101	−0.930	0.366	
	Season (warm): Age (sub-adult)	−0.002	−0.146	0.141	0.074	−0.028	0.978	
	Season (warm): Sex (male)	−0.096	−0.215	0.024	0.062	−1.554	0.123	
	Age (sub-adult): Sex (male)	0.227	0.017	0.438	0.103	2.211	0.039	*
KDE 50%	(Intercept)	2.977	2.805	3.149	0.088	33.640	0.000	*
	Season (warm)	0.305	0.140	0.470	0.085	3.577	0.001	*
	Age (sub-adult)	0.205	0.000	0.409	0.100	2.045	0.054	
	Sex (male)	−0.073	−0.315	0.168	0.116	−0.630	0.538	
	Season (warm): Age (sub-adult)	−0.112	−0.289	0.065	0.092	−1.224	0.223	
	Season (warm): Sex (male)	−0.134	−0.281	0.013	0.076	−1.763	0.080	
	Age (sub-adult): Sex (male)	0.245	0.006	0.483	0.117	2.093	0.049	*

**Table 2:** Linear mixed models that evaluate how the flight distances of immature Andean condor individuals can be affected by age (juvenile or sub-adult), sex and season (warm or cold) and the interactions between these variables. The asterisks show statistically significant results.

Model	Variables	Est. Value	Lower	Upper	Std. Error	*t*-Value	*p*-Value	
Hourly Distance	(Intercept)	2.940	2.805	3.075	0.069	42.746	0.000	*
	Season (warm)	−0.098	−0.148	−0.047	0.026	−3.781	0.000	*
	Age (sub-adult)	0.203	0.085	0.322	0.060	3.371	0.001	*
	Sex (male)	−0.093	−0.292	0.106	0.096	−0.968	0.343	
	Season (warm): Age (sub-adult)	−0.015	−0.069	0.040	0.028	−0.527	0.598	
	Season (warm): Sex (male)	0.081	0.037	0.126	0.023	3.567	0.000	*
	Age (sub-adult): Sex (male)	−0.033	−0.199	0.134	0.085	−0.384	0.701	
Daily Distance	(Intercept)	4.528	4.457	4.599	0.036	125.178	0.000	*
	Season (warm)	0.219	0.191	0.248	0.014	15.211	0.000	*
	Age (sub-adult)	0.137	0.081	0.193	0.028	4.808	0.000	*
	Sex (male)	0.038	−0.039	0.115	0.037	1.041	0.312	

## Data Availability

The data analyzed for the study are available from the corresponding author upon reasonable request.

## Supplementary Materials

The following are available online at https://www.mdpi.com/article/10.3390/ani13071234/s1, Figure S1: Trend throughout the year of home range size for MCP and KDE99% contour area home range estimators, Figure S2: Differences between sexes and ages by season for the logarithm of MCP and KDE99% contour area home range, Table S1: Details of tagged immature Andean condors, Table S2: Description of information and movement parameters of each immature condor tagged with GPS devices, Table S3: Description of information and movement parameters of each immature condor tagged by season, Table S4: Linear mixed models evaluating how home range size of immature Andean condor individuals can be affected by age, sex and season.

## References

[1] Bowler D.E., Benton T.G. (2005). Causes and Consequences of Animal Dispersal Strategies: Relating Individual Behaviour to Spatial Dynamics. Biol. Rev..

[2] Greenwood P.J. (1980). Mating Systems, Philopatry and Dispersal in Birds and Mammals. Anim. Behav..

[3] Ronce O. (2007). How Does It Feel to Be like a Rolling Stone? Ten Questions about Dispersal Evolution. Annu. Rev. Ecol. Evol. Syst..

[4] Gil J.A., Díez Ó. (1993). Dispersión Juvenil Del Quebrantahuesos En Los Pirineos [Juvenile Dispersal of Bearded Vulture in the Pyrenees]. Quercus.

[5] Walls S.S., Kenward R.E. (1998). Movements of Radio-Tagged Buzzards *Buteo buteo* in Early Life. IBIS.

[6] Forsman E.D., Anthony R.G., Reid J.A., Loschl P.J., Sovern S.G., Taylor M., Biswell B.L., Ellingson A., Meslow E.C., Miller G.S. (2002). Natal and Breeding Dispersal of Northern Spotted Owls. Wildl. Monogr..

[7] Snyder N.F., Schmitt N.J., Poole A., Gill F. (2002). California Condor: *Gymnogyps californianus*. The Birds of North America.

[8] Vasilakis D.P., Poirazidis K.S., Elorriaga J.N. (2008). Range Use of a Eurasian Black Vulture (*Aegypius monachus*) Population in the Dadia-Lefkimi-Soufli National Park and the Adjacent Areas, Thrace, NE Greece. J. Nat. Hist..

[9] Alarcón P.A.E., Lambertucci S.A. (2018). A Three-Decade Review of Telemetry Studies on Vultures and Condors. Mov. Ecol..

[10] McCaslin H.M., Caughlin T.T., Heath J.A. (2020). Long-Distance Natal Dispersal Is Relatively Frequent and Correlated with Environmental Factors in a Widespread Raptor. J. Anim. Ecol..

[11] Penteriani V., Ferrer M., Delgado M.d.M. (2011). Floater Strategies and Dynamics in Birds, and Their Importance in Conservation Biology: Towards an Understanding of Nonbreeders in Avian Populations. Anim. Conserv..

[12] Cagnacci F., Boitani L., Powell R.A., Boyce M.S. (2010). Animal Ecology Meets GPS-Based Radiotelemetry: A Perfect Storm of Opportunities and Challenges. Philos. Trans. R. Soc. B.

[13] Penteriani V., Delgado M.d.M. (2009). Thoughts on Natal Dispersal. J. Raptor Res..

[14] Krüger S., Reid T., Amar A. (2014). Differential Range Use between Age Classes of Southern African Bearded Vultures *Gypaetus barbatus*. PLoS ONE.

[15] Margalida A., Pérez-García J.M., Afonso I., Moreno-Opo R. (2016). Spatial and Temporal Movements in Pyrenean Bearded Vultures (*Gypaetus barbatus*): Integrating Movement Ecology into Conservation Practice. Sci. Rep..

[16] Morant J., Arrondo E., Sánchez-Zapata J.A., Donázar J.A., Cortés-Avizanda A., De La Riva M., Blanco G., Martínez F., Oltra J., Carrete M. (2023). Large-Scale Movement Patterns in a Social Vulture Are Influenced by Seasonality, Sex, and Breeding Region. Ecol. Evol..

[17] Morrison J.L., Wood P.B. (2009). Broadening Our Approaches to Studying Dispersal in Raptors. J. Raptor Res..

[18] Clobert J., Le Galliard J.-F., Cote J., Meylan S., Massot M. (2009). Informed Dispersal, Heterogeneity in Animal Dispersal Syndromes and the Dynamics of Spatially Structured Populations. Ecol. Lett..

[19] Clobert J., Baguette M., Benton T.G., Bullock J.M. (2012). Dispersal Ecology and Evolution.

[20] Walters J.R. (2000). Dispersal Behavior: An Ornithological Frontier. Condor.

[21] Dingemanse N.J., Both C., Van Noordwijk A.J., Rutten A.L., Drent P.J. (2003). Natal Dispersal and Personalities in Great Tits (*Parus major*). Proc. R. Soc. London. Ser. B Biol. Sci..

[22] Aebischer A., Nyffeler P., Arlettaz R. (2010). Wide-Range Dispersal in Juvenile Eagle Owls (*Bubo bubo*) across the European Alps Calls for Transnational Conservation Programmes. J. Ornithol..

[23] Delgado M.d.M., Penteriani V., Nams V.O. (2009). How Fledglings Explore Surroundings from Fledging to Dispersal. A Case Study with Eagle Owls *Bubo bubo*. Ardea.

[24] Kouba M., Bartoš L., Štastný K. (2013). Differential Movement Patterns of Juvenile Tengmalms Owls (*Aegolius funereus*) during the Post-Fledging Dependence Period in Two Years with Contrasting Prey Abundance. PLoS ONE.

[25] Nicolaus M., Wang X., Lamers K.P., Ubels R., Both C. (2022). Unravelling the Causes and Consequences of Dispersal Syndromes in a Wild Passerine. Proc. R. Soc. B Biol. Sci..

[26] Ruiz M.D.M., Ramsden D., Roper S., Cresswell B., Skuse J. (2021). Juvenile Barn Owl *Tyto alba* Dispersal: A Radio-Tracking Study of Roost Site Selection in Relation to Landscape Features. Bird Study.

[27] del Hoyo J., Elliot A., Sargatal J. (1994). Handbook of the Birds of the World. New World Vultures to Guineafowl.

[28] Bustamante J., Hiraldo F. (1989). Post-Fledging Dependence Period and Maturation of Flight Skills in the Black Kite *Milvus migrans*. Bird Study.

[29] Bustamante J., Negro J.J. (1994). The Post-Fledging Dependence Period in the Lesser Kestrel *Falco naumanni*. J. Raptor Res..

[30] Krüger S., Amar A. (2017). Insights into Post-Fledging Dispersal of Bearded Vultures *Gypaetus barbatus* in Southern Africa from GPS Satellite Telemetry. Bird Study.

[31] Williams H.J., Shepard E.L.C., Holton M.D., Alarcón P.A.E., Wilson R.P., Lambertucci S.A. (2020). Physical Limits of Flight Performance in the Heaviest Soaring Bird. Proc. Natl. Acad. Sci. USA.

[32] Bamford A.J., Diekmann M., Monadjem A., Mendelsohn J. (2007). Ranging Behaviour of Cape Vultures *Gyps coprotheres* from an Endangered Population in Namibia. Bird Conserv. Int..

[33] Plaza P.I., Lambertucci S.A. (2020). Ecology and Conservation of a Rare Species: What Do We Know and What May We Do to Preserve Andean Condors?. Biol. Conserv..

[34] Birdlife International *Vultur Gryphus*. The IUCN Red List of Threatened Species 2020: E.T22697641A181325230.

[35] Guido J.M., Alarcón P.A.E., Donázar J.A., Hiraldo F., Lambertucci S.A. (2019). The Use of Biosphere Reserves by a Wide-Ranging Avian Scavenger Indicates Its Significant Potential for Conservation. Environ. Conserv..

[36] Cabrera A.L. (1976). Regiones Fitogeográficas Argentinas.

[37] Paruelo J.M., Beltran A., Jobbagy E., Sala O.E., Golluscio R.A. (1998). The Climate of Patagonia: General Patterns and Controls on Biotic Processes. Ecol. Austral.

[38] Ballejo F., Lambertucci S.A., Trejo A., De Santis L.J. (2018). Trophic Niche Overlap among Scavengers in Patagonia Supports the Condor-Vulture Competition Hypothesis. Bird Conserv. Int..

[39] Lambertucci S.A., Navarro J., Zapata J.A.S., Hobson K.A., Alarcón P.A., Wiemeyer G., Blanco G., Hiraldo F., Donázar J.A. (2018). Tracking Data and Retrospective Analyses of Diet Reveal the Consequences of Loss of Marine Subsidies for an Obligate Scavenger, the Andean Condor. Proc. R. Soc. B.

[40] Lambertucci S.A., Trejo A., Di Martino S., Sánchez-Zapata J.A., Donázar J.A., Hiraldo F. (2009). Spatial and Temporal Patterns in the Diet of the Andean Condor: Ecological Replacement of Native Fauna by Exotic Species. Anim. Conserv..

[41] Ferguson-Lees J., Christie D.A. (2001). Raptors of the World.

[42] Wallace R.B., Reinaga A., Piland N., Piana R., Vargas F.H., Zegarra R.E., Alvarado S., Kohn S., Lambertucci S.A., Alarcón P. (2022). Defining Spatial Conservation Priorities for the Andean Condor (*Vultur gryphus*). J. Raptor Res..

[43] Lambertucci S.A., Mastrantuoni O.A. (2008). Breeding Behavior of a Pair of Free-Living Andean Condors. J. Field Ornithol..

[44] Lambertucci S.A. (2007). Biología y Conservación Del Cóndor Andino (*Vultur gryphus*) En Argentina [Biology and Conservation of the Andean Condor (*Vultur gryphus*) in Argentina]. El Hornero.

[45] Alarcón P.A., Morales J.M., Donázar J.A., Sánchez-Zapata J.A., Hiraldo F., Lambertucci S.A. (2017). Sexual-Size Dimorphism Modulates the Trade-off between Exploiting Food and Wind Resources in a Large Avian Scavenger. Sci. Rep..

[46] Perrig P.L., Lambertucci S.A., Donadio E., Padró J., Pauli J.N. (2019). Monitoring Vultures in the 21st Century: The Need for Standardized Protocols. J. Appl. Ecol..

[47] Wallace M.P., Temple S.A. (1988). Impacts of the 1982–1983 El Niño on Population Dynamics of Andean Condors in Peru. Biotropica.

[48] Hofman M.P.G., Hayward M.W., Heim M., Marchand P., Rolandsen C.M., Mattisson J., Urbano F., Heurich M., Mysterud A., Melzheimer J. (2019). Right on Track? Performance of Satellite Telemetry in Terrestrial Wildlife Research. PLoS ONE.

[49] R Core Team (2019). R: A Language and Environment for Statistical Computing.

[50] Fieberg J., Börger L. (2012). Could You Please Phrase “Home Range” as a Question?. J. Mammal..

[51] Walter W.D., Onorato D.P., Fischer J.W. (2015). Is There a Single Best Estimator? Selection of Home Range Estimators Using Area-under-the-Curve. Mov. Ecol..

[52] Bivand R., Keitt T., Rowlingson B., Pebesma E., Sumner M., Hijmans R., Rouault E., Bivand M.R. Package ‘Rgdal.’ Bindings for the Geospatial Data Abstraction Library. https://cran.r-project.org/web/packages/rgdal/index.html.

[53] Calenge C. (2011). Home Range Estimation in R: The AdehabitatHR Package.

[54] Laver P. (2005). ABODE: Kernel Home Range Estimation for ArcGIS, Using VBA and ArcObjects. User Man. Beta Version.

[55] Laver P.N., Kelly M.J. (2008). A Critical Review of Home Range Studies. J. Wildl. Manag..

[56] Powell R.A., Mitchell M.S. (2012). What Is a Home Range?. J. Mammal..

[57] Zuur A., Ieno E.N., Walker N., Saveliev A.A., Smith G.M. (2009). Mixed Effects Models and Extensions in Ecology with R.

[58] Pinheiro J., Bates D. (2000). Mixed-Effects Models in S and S-PLUS.

[59] Alarcón P.A.E. (2016). Movimiento Animal y Patrones Emergentes de Uso Del Espacio: Hacia Una Interpretación Mecanística de La Ecología Del Cóndor Andino (Vultur gryphus) [Animal Movement and Emerging Patterns of Space Use: Towards a Mechanistic Interpretation of the Ecology of the Andean Condor (Vultur gryphus)].

[60] Lambertucci S.A., Alarcón P.A., Hiraldo F., Sanchez-Zapata J.A., Blanco G., Donázar J.A. (2014). Apex Scavenger Movements Call for Transboundary Conservation Policies. Biol. Conserv..

[61] López-López P., Gil J.A., Alcántara M. (2014). Post-Fledging Dependence Period and Onset of Natal Dispersal in Bearded Vultures (*Gypaetus barbatus*): New Insights from GPS Satellite Telemetry. J. Raptor Res..

[62] Martens F.R., Pfeiffer M.B., Downs C.T., Venter J.A. (2018). Post-Fledging Movement and Spatial Ecology of the Endangered Cape Vulture (*Gyps coprotheres*). J. Ornithol..

[63] Kane A., Wolter K., Neser W., Kotze A., Naidoo V., Monadjem A. (2016). Home Range and Habitat Selection of Cape Vultures *Gyps coprotheres* in Relation to Supplementary Feeding. Bird Study.

[64] Margalida A., Carrete M., Hegglin D., Serrano D., Arenas R., Donázar J.A. (2013). Uneven Large-Scale Movement Patterns in Wild and Reintroduced Pre-Adult Bearded Vultures: Conservation Implications. PLoS ONE.

[65] Phipps W.L., Willis S.G., Wolter K., Naidoo V. (2013). Foraging Ranges of Immature African White-Backed Vultures (*Gyps africanus*) and Their Use of Protected Areas in Southern Africa. PLoS ONE.

[66] Li X.-Y., Kokko H. (2019). Sex-Biased Dispersal: A Review of the Theory. Biol. Rev..

[67] Trochet A., Courtois E.A., Stevens V.M., Baguette M., Chaine A., Schmeller D.S., Clobert J., Wiens J.J. (2016). Evolution of Sex-Biased Dispersal. Q. Rev. Biol..

[68] Rivers J.W., Johnson J.M., Haig S.M., Schwarz C.J., Burnett L.J., Brandt J., George D., Grantham J. (2014). An Analysis of Monthly Home Range Size in the Critically Endangered California Condor *Gymnogyps californianus*. Bird Conserv. Int..

[69] Donázar J.A., Travaini A., Ceballos O., Rodríguez A., Delibes M., Hiraldo F. (1999). Effects of Sex-Associated Competitive Asymmetries on Foraging Group Structure and Despotic Distribution in Andean Condors. Behav. Ecol. Sociobiol..

[70] Marinero N.V., Cailly-Arnulphi V.B., Lambertucci S.A., Borghi C.E. (2018). Pigmentation and Not Only Sex and Age of Individuals Affects Despotism in the Andean Condor. PLoS ONE.

[71] Padró J., Pauli J.N., Perrig P.L., Lambertucci S.A. (2019). Genetic Consequences of Social Dynamics in the Andean Condor: The Role of Sex and Age. Behav. Ecol. Sociobiol..

[72] Morales J.M., Moorcroft P.R., Matthiopoulos J., Frair J.L., Kie J.G., Powell R.A., Merrill E.H., Haydon D.T. (2010). Building the Bridge between Animal Movement and Population Dynamics. Philos. Trans. R. Soc. B Biol. Sci..

[73] Nathan R. (2008). An Emerging Movement Ecology Paradigm. Proc. Natl. Acad. Sci. USA.

[74] Buechley E.R., McGrady M.J., Çoban E., Şekercioğlu Ç.H. (2018). Satellite Tracking a Wide-Ranging Endangered Vulture Species to Target Conservation Actions in the Middle East and East Africa. Biodivers. Conserv..

[75] Kang J.-H., Hyun B.-R., Kim I.K., Lee H., Lee J.-K., Hwang H.-S., Eom T.-K., Rhim S.-J. (2019). Movement and Home Range of Cinereous Vulture *Aegypius monachus* during the Wintering and Summering Periods in East Asia. Turk. J. Zool..

[76] Peshev H., Stoynov E., Parvanov D., Grozdanov A. (2018). Seasonal and Spatial Dynamics of the Population of the Griffon Vulture *Gyps fulvus* (Hablizl, 1783) (Aves: Accipitridae) in Southwestern Bulgaria. Acta Zool. Bulg..

[77] Sherub S., Fiedler W., Duriez O., Wikelski M. (2017). Bio-Logging, New Technologies to Study Conservation Physiology on the Move: A Case Study on Annual Survival of Himalayan Vultures. J. Comp. Physiol. A.

[78] Harel R., Duriez O., Spiegel O., Fluhr J., Horvitz N., Getz W.M., Bouten W., Sarrazin F., Hatzofe O., Nathan R. (2016). Decision-Making by a Soaring Bird: Time, Energy and Risk Considerations at Different Spatio-Temporal Scales. Philos. Trans. R. Soc. B.

[79] Urios V., López-López P., Limiñana R., Godino A. (2010). Ranging Behaviour of a Juvenile Bearded Vulture (*Gypaetus barbatus meridionalis*) in South Africa Revealed by GPS Satellite Telemetry. Ornis Fenn..

[80] Perrig P.L., Lambertucci S.A., Cruz J., Alarcón P.A.E., Plaza P.I., Middleton A.D., Blanco G., Sánchez-Zapata J.A., Donázar J.A., Pauli J.N. (2020). Identifying Conservation Priority Areas for the Andean condor in Southern South America. Biol. Conserv..

[81] Arrondo E., Moleón M., Cortés-Avizanda A., Jiménez J., Beja P., Sánchez-Zapata J.A., Donázar J.A. (2018). Invisible Barriers: Differential Sanitary Regulations Constrain Vulture Movements across Country Borders. Biol. Conserv..

[82] Pauli J.N., Donadio E., Lambertucci S.A. (2018). The Corrupted Carnivore: How Humans Are Rearranging the Return of the Carnivore-Scavenger Relationship. Ecology.

