# Supplementary material for: The Influence of Age, Sex and Season on Andean Condor Ranging Behavior during the Immature Stage
Source: Animals (Basel). 2023 Apr 2;13(7):1234. doi: 10.3390/ani13071234 (PMC10092982; doi:10.3390/ani13071234)

Supplementary Material for

Influence of age, sex and season on Andean condor ranging behavior during the immature stage

Jorgelina María Guido[1,2*]; Nicolás Rodolfo Cecchetto[3]; Pablo Ignacio Plaza[1]; José Antonio Donázar[4] and Sergio Agustín Lambertucci[1]

Supplementary Table S1: Details of tagged immature Andean condors. Reported for each bird: ID, device used, capture date and the date of the last connection of the device.

ID	Device	Capture date	Last connection
BLANCA	CTT®-1000-BT3-Series	13/01/2018	15/11/2020
BM27B3_a	NT-VKT	18/12/2013	15/01/2014
BM27B3_b	NT-VKT	14/11/2014	06/07/2016
BPAZIT	NT-VKT	26/10/2013	03/12/2013
CHITA	CTT®-1000-BT3-Series	13/01/2018	18/11/2020
CT4072	CTT®-1090	06/12/2011	15/03/2016
CT8654	CTT®-1090	26/10/2014	02/01/2014
CT9058	CTT®-1090	19/01/2014	16/03/2014
CT9563	CTT®-1090	04/11/2013	06/06/2015
DCJ0MT	NT-VKT	26/10/2013	07/04/2016
HECTOR	CTT®-1000-BT3-Series	13/01/2018	13/11/2020
IHWEY8	NT-VKT	04/11/2013	02/01/2014
JA5W9H	NT-VKT	18/12/2013	30/12/2013
N6M1M6	NT-VKT	02/11/2013	20/04/2014
NT113CZS	NT-VKT	26/10/2013	13/11/2013
NT12VG3Y	NT-VKT	18/12/2013	19/01/2014
O4WOMJ	NT-VKT	02/11/2013	14/01/2017
PEPO	CTT®-1000-BT3-Series	25/01/2018	08/11/2020
RK2GX6	NT-VKT	02/11/2013	04/12/2013
TOTO	CTT®-1000-BT3-Series	27/01/2018	06/12/2020
TP1PYG	NT-VKT	18/12/2013	08/01/2014
TWFXZJ	NT-VKT	02/11/2013	16/01/2014
U0MLYR	NT-VKT	29/10/2013	17/12/2015
VFE3F9	NT-VKT	29/10/2013	05/11/2013
VHE95O	NT-VKT	29/10/2013	03/11/2013
VHSRLQ	NT-VKT	19/11/2014	04/12/2014

Supplementary Table S2: Description of natural history information and movement parameters of each immature condor tagged with GPS devices. For each individual we report the ID, sex, age, total number of sampled months (N° total month) and total number of GPS locations for the total months (N° total locs.). In addition, we report the number of months that reached more than 140 locations per month (Months + 140 locs.) and were used in the home range analyses, and their respective number of GPS locations to those are reported (N° locs. (month +140 locs.)). Moreover, the home range areas of the maximum flight distances of each marked immature Andean Condor are reported. The MCP of 100%, the KDE of 99%, 95% and 50% of the contour area are reported, as well as the maximum distance between two successive locations separated by a time interval of one hour (Max. dist. x hr), and the maximum distance flown in one day (Max. daily dist.), as well as the total latitudinal (North-South) and longitudinal (West - East) distance flown. In grey, the individuals that did not reach the minimum values of GPS locations necessary to be considered in the statistical analyses.

ID	Sex	Age	N° total month	N° total locs.	Month + 140 locs.	N° locs (month +140 locs)	MCP (km^2)	KDE 99% (km^2)	KDE 95% (km^2)	KDE 50% (km^2)	Max. dist. x hr (km)	Max. daily dist. (km)	Total lat. dist. (km)	Total long. dist. (km)
BLANCA	F	SA	35	6012	16	4855	91,445.8	63,136.6	43,154.3	6500.2	94.6	238.5	636.6	214.0
BM27B3_a	M	SA	2	284	1	148	15,416.4	18,021.4	12,957.6	2714.1	48.2		299.4	75.0
BM27B3_b	F	JUV/SA	22	2950	8	1835	102,073.9	82,475.5	58,703.3	10,749.9	58.4	139.2	811.9	177.0
BPAZIT	H	SA	3	230	1	186	21,151.3	21,212.7	15,384.3	3125.2	44.5	167.7	269.9	123.6
CHITA	F	JUV/SA	35	13,034	33	12,855	103,598.4	67,184.0	45,085.5	7364.1	65.2	279.5	675.9	203.9
CT4072	M	SA	13	1766	4	1035	127,960.6	77,777.8	53,485.7	7047.6	89.6	196.0	853.8	256.7
CT8654	M	JUV	4	954	2	854	15,386.6	14,788.3	9290.8	1059.0	53.2	197.7	162.4	138.6
CT9058	M	SA	3	520	2	434	8896.8	10,635.0	7389.3	1400.7	60.6	173.1	109.2	166.6
CT9563	M	JUV/SA	16	2802	8	2306	114,371.6	52,356.2	31,861.0	4588.9	62.7	257.3	896.0	239.1
DCJ0MT	F	SA	31	5815	21	4821	62,433.4	45,926.6	31,351.4	5649.2	54.1	209.9	422.2	205.9
HECTOR	M	SA	33	4783	12	3857	189,888.8	114,995.2	72,668.1	6749.9	118.7	302.2	1351.5	209.2
IHWEY8	F	SA	3	298	1	188	20,962.6	21,621.9	15,073.9	2121.9	47.4		261.6	124.1
JA5W9H	M	SA	1	128			7989.3	10,886.6	7727.4	1758.3	33.6	119.8	193.1	66.3
N6M1M6	M	SA	6	1137	5	1057	34,989.3	25,677.1	15,306.6	3066.8	57.8	213.1	493.7	133.3
NT113CZS	F	SA	2	57			11,459.2	10,927.4	7557.4	1384.9	26.3		221.8	78.0
NT12VG3Y	M	JUV	2	160			6465.0	8768.2	5752.6	1048.1	36.7	94.9	86.0	96.6

Supplementary Table S2 (continuation): Description of natural history information and movement parameters of each immature condor tagged with GPS devices. For each individual we report the ID, sex, age, total number of sampled months (N° total month) and total number of GPS locations for the total months (N° total locs.). In addition, we report the number of months that reached more than 140 locations per month (Months + 140 locs.) and were used in the home range analyses, and their respective number of GPS locations to those are reported (N° locs. (month +140 locs.)). Moreover, the home range areas of the maximum flight distances of each marked immature Andean condor are reported. The MCP of 100%, the KDE of 99%, 95% and 50% of the contour area are reported, as well as the maximum distance between two successive locations separated by a time interval of one hour (Max. dist. x hr), and the maximum distance flown in one day (Max. daily dist.), as well as the total latitudinal (North-South) and longitudinal (West - East) distance flown. In grey, the individuals that did not reach the minimum values of GPS locations necessary to be considered in the statistical analyses.

ID	Sex	Age	N° total month	N° total locs.	Month + 140 locs.	N° locs (month +140 locs)	MCP (km^2)	KDE 99% (km^2)	KDE 95% (km^2)	KDE 50% (km^2)	Max. dist. x hr (km)	Max. daily dist. (km)	Total lat. dist. (km)	Total long. dist. (km)
O4WOMJ	F	SA	36	6600	23	5547	70,997.2	56,892.0	40,104.6	6713.5	63.8	233.1	656.0	164.1
PEPO	M	SA	30	5385	13	4563	141,050.3	91,317.6	61,730.5	11,609.0	68.3	274.3	986.4	225.9
RK2GX6	F	SA	2	242	1	216	43,958.7	33,094.2	22,856.6	3647.7	43.8	134.5	536.7	110.5
TOTO	M	JUV/SA	35	8858	22	7381	227,162.4	100,520.3	55,572.7	5961.6	110.6	270.4	1261.9	291.6
TP1PYG	F	JUV	2	66			8092.1	8606.6	5913.9	746.0	32.2		237.3	73.5
TWFXZJ	M	SA	3	215			59,449.6	40,211.2	27,884.2	4480.7	42.1	88.4	556.6	145.5
U0MLYR	F	JUV/SA	19	2689	8	1953	55,951.8	46,456.4	33,812.7	7598.0	52.9	189.7	519.8	145.3
VFE3F9	F	SA	1	19			1206.1	4761.7	3170.4	541.1	47.8		61.9	81.6
VHE95O	F	SA	1	9			613.0	3873.3	2596.1	502.6	24.4		60.0	17.9
VHSRLQ	F	JUV	2	177			11,435.6	12,653.5	8791.8	1864.9	35.3	141.3	221.1	81.6
		**Total**	**342**	**65,190**	**181**	**54,091**	**438,260.0**	**206,854.7**	**121,812.1**	**15,282.9**			**1969.7**	**385.4**
		**Maximum**					**227,162.4**	**114,995.2**	**72,668.1**	**11,609.0**	**118.7**	**302.2**	**1351.5**	**291.6**

Supplementary Table S3: Description of natural history information and movement parameters of each immature condor tagged with GPS devices. For each individual we report the ID, sex, age, and for each season we report the number of months that reached more than 140 locations per month (N° months + 140 locs.) and number of GPS locations for the months that reached more than 140 locations (N° locs. (month + 140 locs.)

			Warm season		Cold season	
**ID**	**Sex**	**Age**	**N° month + 140 locs.**	**N° locs. (month +140 locs)**	**Month + 140 locs.**	**N° locs (month +140 locs)**
BLANCA	F	SA	9	2946	7	1909
BM27B3_a	M	SA	1	148		
BM27B3_b*	F	JUV	5	1245	1	213
BM27B3_b*	F	SA	2	377		
BPAZIT	F	SA	1	186		
CHITA*	F	JUV	6	2006	6	2662
CHITA*	F	SA	10	3228	11	4959
CT4072	M	SA	3	820	1	215
CT8654	M	JUV	2	854		
CT9058	M	SA	2	434		
CT9563*	M	JUV	4	1239	1	273
CT9563*	M	SA	1	340	2	454
DCJ0MT	F	SA	14	3451	7	1370
HECTOR	M	SA	8	2697	4	1160
IHWEY8	F	SA	1	188		
N6M1M6	M	SA	5	1057		
O4WOMJ	F	SA	8	1587	15	3960
PEPO	M	SA	5	1424	8	3139
RK2GX6	F	SA	1	216		
TOTO*	M	JUV	5	2078	4	1054
TOTO*	M	SA	9	3132	4	1117
U0MLYR*	F	JUV	4	1162	2	388
U0MLYR*	F	SA	1	187	1	216

*same individual at different ages

Supplementary Table S4: Linear mixed models that evaluate how home range size –100% Minimum Convex Polygon (MCP) and Kernel Density Estimator (KDE) 99% of the contour area – of immature Andean condor individuals can be affected by age (juvenile or sub-adult), sex and season (warm or cold). The asterisk shows statistically significant results.

Model	Variables	Est. value	Lower	Upper	Std. Error	t-value	p-value	
**MCP**	(Intercept)	3.845	3.660	4.029	0.094	40.755	0.000	*
	Season(warm)	0.325	0.231	0.418	0.048	6.784	0.000	*
	Age(sub-adult)	0.190	0.007	0.372	0.089	2.137	0.045	*
	Sex(male)	0.032	-0.139	0.203	0.082	0.395	0.698	
**KDE 99%**	(Intercept)	3.971	3.860	4.083	0.057	69.487	0.000	*
	Season(warm)	0.204	0.145	0.263	0.030	6.716	0.000	*
	Age(sub-adult)	0.157	0.052	0.263	0.051	3.072	0.006	*
	Sex(male)	0.003	-0.105	0.111	0.052	0.052	0.959	

Supplementary Figure S1: Trend throughout the year of home range size for different home range estimators: A) 100% Minimum Convex Polygon and B) the Kernel Density Estimator (KDE) of 99% of the contour area.

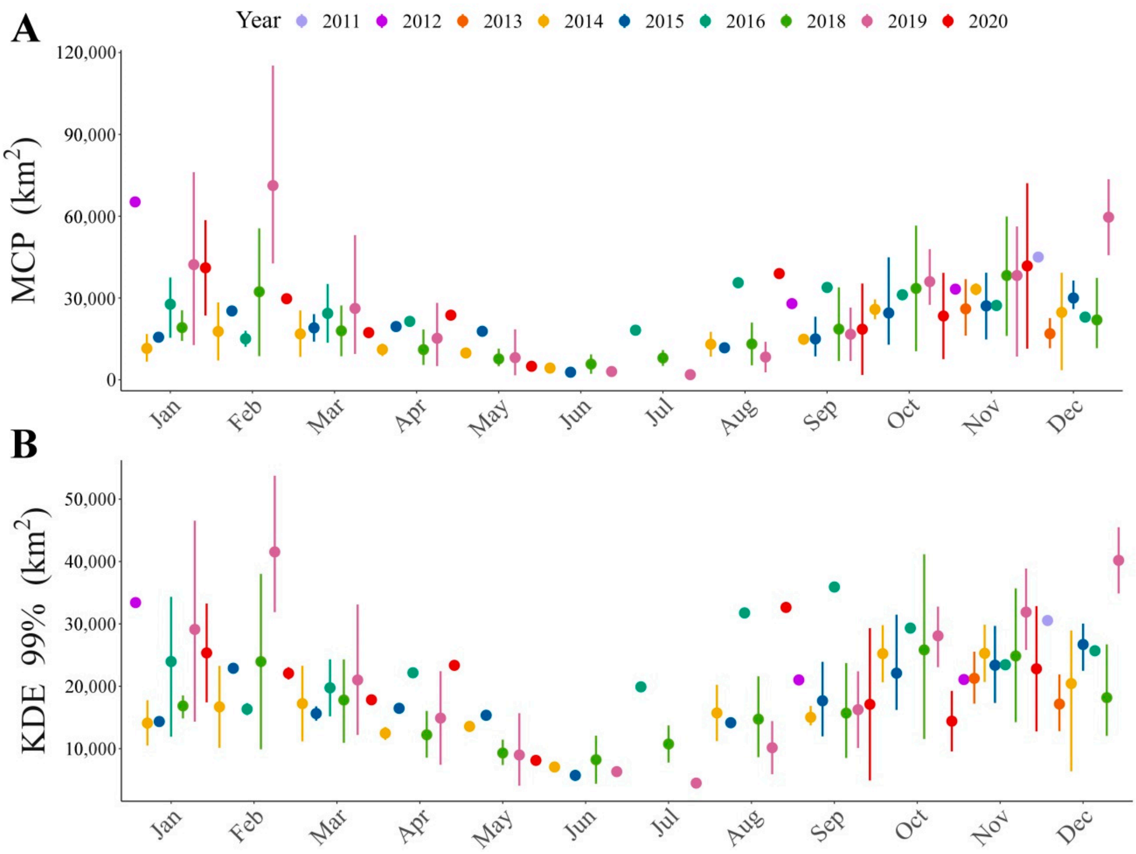

Supplementary Figure S2: Differences between sexes and ages by season for the logarithm of the different estimators: A) 100% Minimum Convex Polygon and B) the Kernel Density Estimator (KDE) of 99% of the contour area.

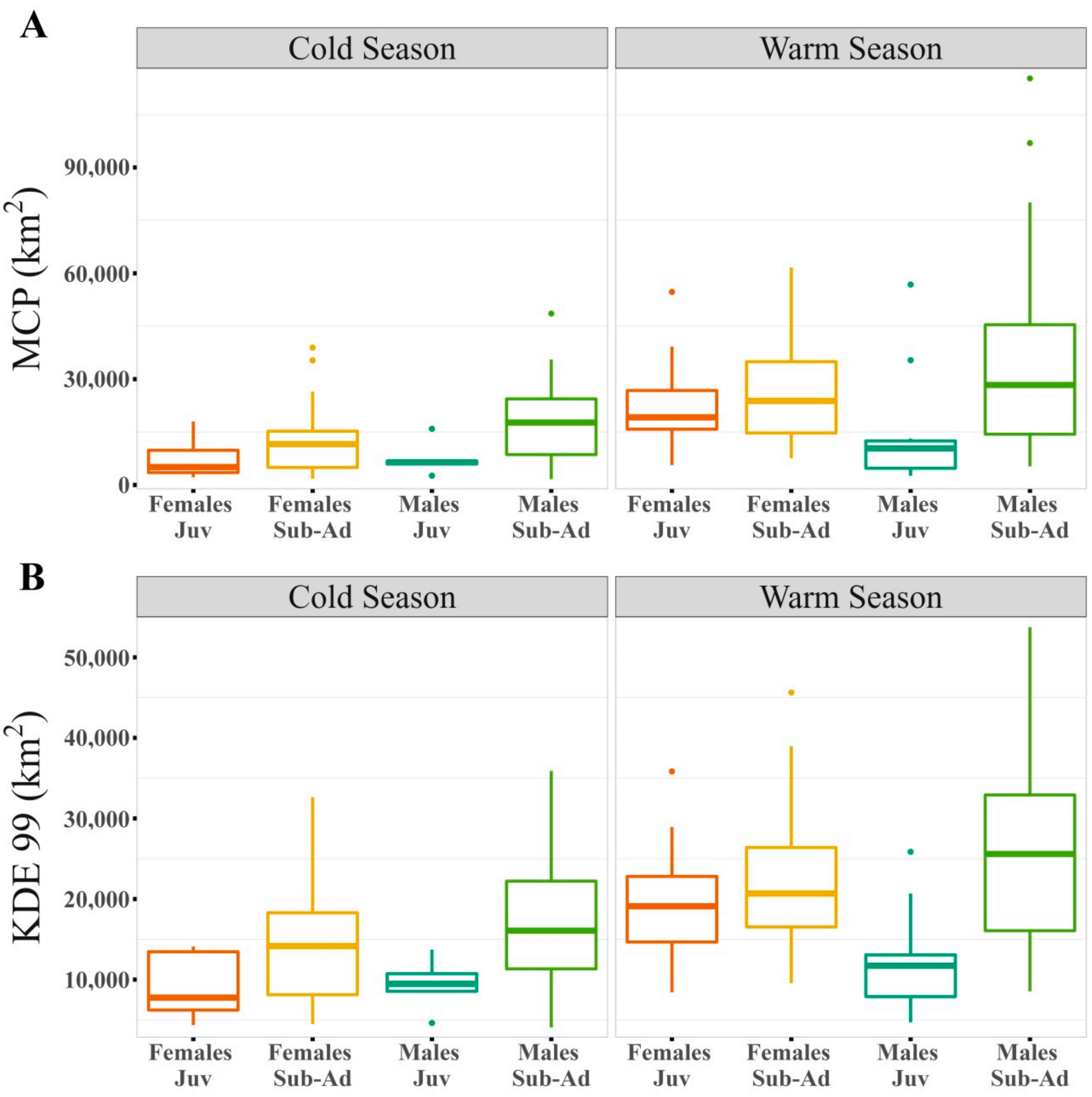

Supplement: Supplementary file 1 [file animals-13-01234-s001.zip › animals-2299030-supplementary.pdf]
